# Developing paediatric eye care teams in India

**DOI:** 10.4103/0301-4738.60082

**Published:** 2010

**Authors:** Ramesh Murthy, Giridhar Pyda, Rohit C Khanna, G V Rao

**Affiliations:** Department of Paediatric Ophthalmology and Strabismus, Oculoplasty and Ocular Oncology, L V Prasad Eye Institute, Hyderabad, India; 1 International Centre for the Advancement of Rural Eye Care, L V Prasad Eye Institute, Hyderabad, India; 2 Orbis International, A-8, Institutional Area, Karkardooma, New Delhi, India

Dear Editor,

Tackling childhood blindness is a priority for many reasons - the number of blind years a child has to face is much more than an adult and, secondly, many causes of childhood blindness are preventable or treatable.[1] Of the approximately 1.4 million children who are blind, about three-quarters live in the poorest countries of Asia and Africa.[2] It is estimated that the prevalence of childhood blindness in India is about 1 per 1000 children.[3] The VISION 2020 - Right to Sight initiative considers control of childhood blindness as one of the priority areas.

Examining children needs special skills and the treatment requires specific training, knowledge and equipment. There are few trained paediatric-oriented ophthalmic personnel in India.[4] In a survey of paediatric eye care facilities in India, opportunities to train a paediatric eye care team was noted to be grossly lacking.[4] The paediatric ophthalmology learning and training center (POLTC) project is an initiative by ORBIS International, an international non-governmental organization, in collaboration with tertiary eye care institutes in India toward the development of comprehensive paediatric eye care teams.

The POLTC project aims at the development of a paediatric eye care team comprising of six personnel: An ophthalmologist, anaesthetist, optometrist, nurse, counsellor and outreach coordinator by building their capacity through specific training in the management of eye diseases in children. Hospitals throughout the country designate teams through ORBIS to the respective tertiary eye care institutes. The ophthalmologist gets training for 12-18 months with a focus on the management of squints, paediatric cataract, amblyopia, corneal problems, retinopathy of prematurity, retinoblastoma management and congenital glaucoma. In addition, it also includes the component of community eye care, especially vision screening and awareness initiatives. Targets and indicators are set for number of children examined, number of children treated, number of children undergoing surgery and number of children receiving spectacles. The project also encompasses capacity building of partner hospitals by providing infrastructure and technical support. Providing various research opportunities and conducting research on topics that have a profound impact on the community is again an integral part of this project. The project also supports regular hospital-based training programs and supports continuing medical education programs that are important to build the capacity of the POLTC staff. Training of all the personnel is performed in established international, acclaimed centers of excellence in India. The teams, after training, have a commitment to serve in the underserved areas of the country or at their sponsoring hospitals and, specifically, tackle children's eye diseases with this newly gained expertise. The activities that are a part of the project are broad based and targeted toward health promotion and awareness creation relating to eye diseases among children and society as a whole. A team approach ensures that service delivery is optimal. Many such teams have already been trained and are working successfully in different parts of India creating awareness on eye diseases in children and providing specialized paediatric eye care. It is hoped that creation of such paediatric eye care teams will have a great impact on the community, but the effects will only be obvious in the times to come.
